# Shaping space. A conceptual framework on the connections between organised crime groups and territories: An introduction to the special issue on ‘Spaces of Organised Crime’

**DOI:** 10.1007/s12117-021-09415-0

**Published:** 2021-04-07

**Authors:** Anna Sergi, Luca Storti

**Affiliations:** 1 Department of Sociology, University of Essex, Wivenhoe Park, Colchester, CO43SQ UK; 2 Dipartimento di Culture, Politica e Società, University of Turin, Turin, Italy

**Keywords:** Organised crime, Space, Place, Criminal networks, Territories, Contexts

## Abstract

This paper, which is the introduction to this special issue on ‘Spaces of Organised Crime’, aims to analyse the nexus between organised crime groups and territories. Such groups are able to exploit resources that circulate within territorial contexts in which they are embedded. They also operate concretely as entities that can take part to the transformation of *spaces* into *places*. Accordingly, we will lay out an analytical model about the processes through which organised crime groups contribute to create and shape *territories*. We show how these processes link with the main types of organised crime groups on a differentiated basis. In the last section of this introduction, we present the papers included in the special issue and the logic connecting them to one another.

## Introduction and background

The issue on “how the territorial context matters” in relation to Organised Crime Groups (hereafter OCGs) is a crucial one independently of the specific type of OCG. Long gone are the days when scholars used to concentrate merely on the internal features of OCGs, without systematically looking at the territorial environment in which they are embedded (Kleemans 2013; Sciarrone 2008; Sergi 2017). Studies integrating considerations of the endogenous aspects of OCGs within territorial contexts have recently grown in number and gained importance (Sciarrone and Storti 2019). Despite being involved in illegal activities and being partially secret organisations, OCGs are also (partly) open systems and, as such, they establish relations with the local societies in which they operate. Needless to say, studies on organisational and endogenous aspects of OCGs have been absolutely relevant. They bring an actual added value whenever they combine the analysis of coordinated actions, types of organisation and membership with the analysis of external social environments. Given these general premises, how can we illustrate the relationships between OCGs and their territorial environments? More precisely, how OCGs affect the 'territorial dimension' of the contexts they are in and how do territories affect OCGs?

To approach these questions, we need to start from the main conceptualisations of the link between OCGs and territorial contexts that are present in the literature. These conceptualisations can be grouped into three main streams.

The first perspective identifies the "territorial context" as a sort of container of opportunities and constraints, push and pull factors (Kleemans and Van de Bunt 2008; Von Lampe 2016). Opportunities are linked with the main characteristics of local politics and economy, while constraints pertain to – among others – the activities of prosecutors and enforcement authorities. Based on this perspective, OCGs are characterised by exchanges of resources with external circles. This is a relevant perspective, but it is might still be limited by a dualistic and schematic vision.

The second perspective establishes a more profound link between OCGs and territorial contexts instead, which is assumed as the ‘second skin’ of OCGs (Hess 1970). This interpretation has been long applied to mafia-type groups, as structured expression of values, normative orientations, and the "common culture" of local societies and territorial areas (Sergi 2018). Even if this perspective has helped frame multi-dimensional aspects of organised crime phenomena, it struggles at times to avoid stereotyped equations between OGCs and their socio-cultural contexts, as if the latter were ontologically criminogenic in character.

The third perspective is somehow more dynamic and holistic in nature. Territorial contexts in which OCGs are active can be seen as "organisational environments": a field made up of institutions, collective and individual actors deeply interconnected between one another. With the concept of "organisational environments", we have also to assume a poly-dimensional idea of embeddedness (Powell and DiMaggio, 2004; DiMaggio and Zukin 1990). OCGs are indeed not only settled in relational structures but also moulded by conventions, shared understandings and convictions, collective representations, that is to say they are also culturally and cognitively embedded at the local level (Sergi 2016; Hobbs and Antonopoulos 2013).

In this paper – as an introduction to the special issue—we argue that OCGs identify, design, build and interpret several ‘spaces’ thus creating or affecting ‘territories’ and ‘social places’. Territories and social places emerge when a space – as substratum – is being socially elaborated and shaped (Duarte 2017; Agnew 2012). In other terms, territories can be conceived as *arenas*, which are socially built and constantly negotiated. The processes of social construction of territories require attention in order to understand the social meaning of *places* located within broad regional areas. Moreover, attention should be paid to the individual and collective actors building this arena. OCGs can be among these actors. To argue this, we depart from a perspective according to which OCGs are embedded in multiple ways (i.e., structural, cognitive, cultural) in territorial contexts thus giving rise to specific organisational and institutional environments. We bring into this field of analysis a multidisciplinary approach, which is mainly rooted in criminology and sociology, and we aim to deepen further the analysis by questioning *how* OCGs shape territories where they exist and operate.

This introduction to the special issue proceeds as follow. The next section deals with a brief overview of how we make sense of OCGs with specific reference to criminal structures and organisations. Afterwards we will identify some of the main processes through which OCGs are involved in the shaping of territories. We will then pinpoint some aspects to reflect on the relationships between the *sense-making of places*, on the one hand, and the characteristics of criminal organisations, on the other. Once the theoretical framework of the introductory paper has been outlined, we will briefly illustrate the individual contributions of the special Issue on “Spaces of Organised Crime”. As we will show, each paper relates to different types of criminal organisations, groups and networks, active in a variety of contexts everywhere on the planet. A broader and transversal perspective like the one we propose makes it possible to illustrate the nexus between OCGs and territories in its different manifestations.

## Types of OCGs and types of territorial connection

**Fig. 1:**
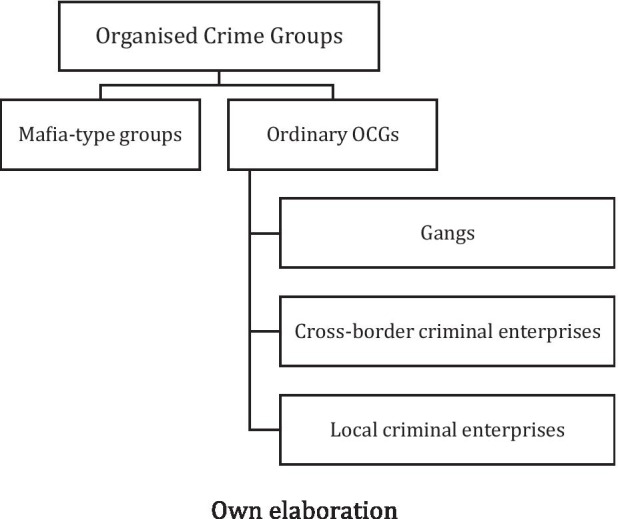
**OCGs: a taxonomy.** Own elaboration

A first way to push forward our analysis is to attempt a distinction across different types of OCGs by applying the logic of a taxonomy (see Fig. 1).

At first stage, we can isolate mafia-type groups from other types of OCGs. We thus agree with claims that mafia groups are a *specific type* of organised crime, being the latter the *species* of a more varied *genus* of organised crime (Sciarrone 2008; Sciarrone and Storti 2014, 2019; Sergi and Storti 2020; Dagnes et al. 2020; Von Lampe 2016). Mafias are not only profit-driven, they are also power-driven (Sergi 2017). They are both power and enterprise syndicates (Block 1980). Varese (2011) postulates that organised crime groups vary through the combination of three functions: producing, trading, and governing (see also Shortland and Varese 2016: 811). *Producing* is mainly about how organised crime groups creates an illegal good (i.e. a type of drugs) or accomplishes a licit goal/service in an illicit way. By definition, *trading* pertains to how OCGs exchange goods in order to gain profit. Lastly, *governing* refers to how OCGs are able to get control over local societies and economy. Aside from these categories, reconstructing how these three functions manifest under concrete cases of OCGs is a task for empirical research. However, we can add a general consideration. Most OCGs are mainly devoted to produce and trade, and less to govern, whereas mafia-type groups have a quintessential orientation towards governing, even though producing and trading feature among their activities too. Indeed, as said, if making a profit is the primary goal of ordinary types of OCGs (Albanese 2000; Von Lampe 2016; Finckenauer 2005), mafia-type groups seek power by embedding themselves in local societies (Sergi 2017; Varese 2011; Sciarrone and Storti 2014). Therefore, the interconnections between mafia-type groups and their territories tend to be poly-dimensional, poly-crime, and denser (Sergi 2017).

At the second stage of the taxonomy, we can add other aspects to distinguish several *types* of OCGs. We can take into account two large themes of the criminological literature to unpack this.

Firstly, analyses of illicit organisations and networks: several scholars have been trying to investigate to what extent members of OCGs are connected with one another and within specific organisations (Morselli 2009; Hughes, Bright and Chalmers, 2017; Calderoni and Superchi, 2019). Indeed, there are studies attempting to look at OCGs as networks, firms and, more generally, business models (Morselli 2009; Hobbs 2001; Catino 2019), with different degrees of agency (Sergi 2020b). Furthermore, some studies of organisational aspects of OCGs have also looked at the reasons why members of OCGs might try to scale up their organisations, in terms of internal complexity, hierarchical structure, membership obligations and choices of activities, including violence (Kleemans and Van Koppen 2020; von Lampe 2016; Hall and Scalia 2019; Catino 2014). These types of OCGs organizations are most commonly indicated in literature as criminal enterprises (von Lampe, 2016; Sergi, 2017). To briefly explore these types of OCGs, we will assume the alternatives between local crime activities and organisations, and non-local ones (Storti, 2018). OCGs might be mainly active in a circumscribed territory where they look for business, but also power and consensus (i.e. local criminal enterprise). OCGs can be also rooted in open global economies, which — by definition — are not restricted to the local level and have a high degree of relational intensity between transnational places (i.e. cross-border criminal enterprise).

Secondly, analyses of the social embeddedness of crime groups: some scholars have observed how some key activities of OCGs and the exercise of their power can manifest at territorial level. This has been done for high-level mafia-type groups, invested both in low level profit-driven activities (i.e. extortion) as well as power-driven ones (such as infiltration in local governments) as well as for other types of OCGs (Sciarrone and Storti 2019; Sergi 2020a, b; Sergi and Storti 2020). In this second perspective also fall, indeed, studies on gangs. Research on gangs is vast and have a long history (Thrasher 1927). Here we recall the street dimensions of gangs, especially youth gangs, and gang-related crimes, that maintain their connection to a given space. Although at times gangs adopt more business orientations and involve older members too, it is in the exploitation of territorial knowledge of the street, neighbourhoods, suburban areas that they evolve (McLean et al 2019).

Overall, OCGs move on a spectrum – an organised crime-mafia spectrum (Sergi, 2017)—that assumes not only that the aims of each of these groups can be different (profit, power); their activities can be different (producing, trading, governing); their structures can be different (networked, hierarchical, horizontal, etc.) and of course, at times they prove to be more successful, at times less. Differently modulated OCGs within a spectrum of structures and activities (to simplify) will interact differently with the territories. Some OCGs will make the territories central to their criminal project: mafia-type groups do this, for political advancement, and so do certain local gangs for reputation and identity. Other OCGs are instead detached from a physical territory as they engage into poly-crime, or non-territorially specific activities: this is the case of systemic corruption/collusion networks, as well as online fraudsters, or groups engaging in financial crimes.

This taxonomy supports the statement that OCGs – alongside other social actors, phenomena and processes – take part to the transformation of spaces into *places* (Chiodelli et al. 2017). In an attempt to simplify all this complexity, without losing any of the nuances of the research, we need to look further at the social construction of space by OCGs.

## OCGs and the social construction of spaces

As already said in this introduction, many OCGs are often space-shaping actors. They identify, design, build several ‘territories’ and ‘social places’, by means of elaborating and shaping territory and space (Duarte 2017; Agnew 2012). This assumption allows to observe them as governance actors as well. Indeed, we trace the shaping of space by OCGs back to four ideal–typical processes that have several aspects in common with governance dynamics:

1. *Controlling the space*: the function of allocating resources to, and manipulating, the space;
2. *Representing the space*: the function of exhibiting power and fostering collective identities;
3. *Diversifying the space*: the further function of structuring and maintaining activities in/through space, both with aims of controlling and with aims of interconnecting actors within it;
4. *(Inter)connecting the space*: the function of creating some sort of link between territorial or non-territorial points that are close or far away to one another.

As for (I) *controlling the space*, we might recall that environmental criminologists have already highlighted how *“the geographic location of various social activities and the architectural arrangements of spaces and building can promote or retard crime rates”* (Gieryn 2000: 480; Brantingham and Brantingham 1990). Notwithstanding the limits of applying situational crime prevention and environmental criminology principles to organised crime (Von Lampe 2011), OCGs can facilitate some crime activities within certain territories as well as keeping other criminal activities under control. For instance, OCGs can reinforce some degree of distinction between blocks and neighbourhoods in large cities when concentrating drug imports in some areas and calming down some visible forms of street crimes, so to avoid high level of police control. While settings related to OCGs appear to be more complex and populated than conventional crime settings (Von Lampe 2011), OCGs can act as both encouragers and discouragers of crimes in certain situations.

As for (II) *representing the space*, OCGs are often involved in creating spatial niches that become relevant social places within territories. Symbolic and subjective values exploited and employed by OCGs demarcate some social places. *Controlling* territories is a matter of trying to establish ‘sovereignty’ over those occupying it; giving symbolic values to places is a matter of soft power and collective identities (Samuel 2013). For instance, street-level OCGs can identify some spots in neighbourhoods to meet with each other and enact their internal hierarchy. More structured groups use ad-hoc places to tie up their membership and enforce collective identities, such as restaurants (Meli 2015) or even religious sanctuaries (Sergi and Lavorgna 2016); this is a function of representation that reinforces OCGs power and structures.

As for (III) *diversifying the space*, OCGs are involved in organising activities within both the licit and illicit economies. In so doing, they can contribute to the functional diversification of areas within territories (Hobbs 1998). They can invest their money in the legal economy by supporting small and medium businesses rooted at the local level (i.e., production clusters). Overall, OCGs put their money in traditional economic sectors having strong connections with territorial resources (i.e., real estate, tourism, commercial activities) (Sciarrone and Storti 2019; Kruisbergen et al. 2015). For example, by exploiting their relational resources, OCGs might influence urbanistic or zoning processes, through which some areas are characterised by a high level of land consumption due to construction activities, and other areas become commercial districts (Chiodelli 2019). In the illegal economy, when OCGs are involved in street-level drug dealing or management of sex work and prostitution, they can shape territorial niches and promote “specific atmospheres” – reputation—in specific urban or suburban areas or districts.

Lastly, as for (IV) *(inter)connecting the space*, some OCGs are able to embed themselves in specific territories as well as connect/interconnect different and at times geographically distant people and places. Accordingly, these OCGs are *glocal phenomena* (Hobbs 1998; Sergi and Lavorgna 2016). Some groups tend to be *mobile* only within circumscribed areas, thus having a lower degree of local embeddedness, but also a scarce capability to expand their radius of action (Sergi 2017). Additionally, some OCGs can *expand* territorially—under certain circumstances—whereby seeking new markets and broadening their business range, even establishing from time to time a partial territorial control in previously ‘immune’ areas (Sciarrone 2008; Morselli et al. 2011; Sciarrone and Storti 2014; Sergi 2019; Varese 2020).

Therefore, we see that OCGs also affect a specific type of spatial *construction*, which has become increasingly important (Castells, 2004; Appadurai, 1996). This is about the creation of territorial networks, the nodes of which are places, and the links between them are “*scapes”* (or flows) of goods and/or persons. Within these transversal spaces, criminal groups establish themselves as multi-sited entities.

## A framework to interpret how OCGs shape space

As we have presented the main components of the framework, we can now move further into the analysis. In Table 1 we consider how different OCGs engage with abovementioned ways of shaping spaces, with a view to build an analytical framework to interpret such relationships. For convenience, and without any claim of completeness, we apply the types of the taxonomy we have defined in the second section (Fig. 1) of this introduction: gangs (street/local); mafia-type; criminal enterprises. As said, criminal enterprises can be further differentiated depending on their activities – in cross-border and local. Certainly criminal enterprises can be hybrid, including tangible goods or services (e.g. drug trafficking) or intangible ones (e.g. money laundering activities done online). And, of course, these ideal types should be read in their simplicity as willing to capture only the main manifestations of criminal structures in territories, while acknowledging a multitude of other situations that might fall out this initial categorisation.

**Table 1:** OCGs type shaping space

	Controlling the Space	Representing the Space	Diversifying the Space	(Inter)Connecting the Space
Mafia-Type	organisation of protection at the local level	use of social space for gathering and collective identity building	ability to exploit the space for various investments and diversification of legal/illegal activities	ability to connect different social groups: relational capacity more or less developed; some mafia clans are mobile and expand in other territories
Gangs (street-local)	manipulation of criminal markets; street location	use of social space for gatherings; demarcation of space		
Cross-border Criminal Enterprise			managing logistics in drug importation and re-investments in legal economy to launder proceeds of crime	different trafficking operations require activities in different territories, more or less far from one another
Local Criminal Enterprise	online fraud manipulating the ‘virtual space’; commercial zoning		fraud followed by money laundering or other exchange of favours	

As stated before, these ideal types and relations are mainly useful to briefly sum up the whole picture of the debate on organised crime and space as we intend it. Therefore, they are neither exclusive nor they represent the ‘conclusive landmark’ of long-term debates in criminological studies. Indeed, our classification of gangs, mafia-type and criminal enterprises, implicitly embodies two main analytical issues: the mechanisms through which OCGs are defined, the main aims (profit/power), and the reach they have (local/global), with specific regard to the territorial level.^1^

With respect to gangs, for instance, the casual paradigm of social specificity – street knowledge—is still assumed to be relevant, for a variety of reasons: the street space not only has boundaries that can be controlled, but also can be given meaning and values as *places*. Furthermore, the origins of gangs are often linked to variables pertaining to social and territorial inequalities, which tend to give rise to segregated and discriminated groups of people according to their social class or their ethnic origin. The lack of opportunities might facilitate the emergence of gangs that are sensitive phenomenon to regional disparities (Durán 2018; Floríndez and Floríndez 2018).

By contrast, criminal enterprises, whether with a more local or a global reach, are based on hypertrophic business opportunities, which have been generated by deregulations of the new liberal era (Ruggiero 2013): their prime characteristic is to discard space boundaries, especially if their activities are online to some extent. In so doing, however, they increase connectivity and succeed in diversifying the spaces they touch. Hence, criminal enterprises can also be seen as a deterritorialised form of organised crime exploiting the lack of legislation concerning financial markets and bank systems through legal or quasi-legal activities that is to be found in several countries (Mugarura 2017).

Mafia-type groups can also be both local and global; the space for them is certainly important as *place* with social meaning at the local level. Some mafia groups have been also reinvigorated by new opportunities in trading and exchanges at the global level, facilitated by globalisation. When mobile, mafia-type groups can also just behave as criminal enterprises, discarding the space dimension far from home. From a theoretical standpoint, mafia-type groups tend primarily to *control* and *organise* places, but they also engage in *representing* and create multiple *interconnections* between close and far away areas, whenever they realise an expansion process.

Finally, it must be highlighted that these typified relationships between OCGs ideal types, and space are useful to strength the connection between analyses of OCGs—their aims, reach and behaviours—and analyses on socio-geographical processes. In doing so, these typified relationships help us situate the debate within different streams of literature (i.e. criminology, economic sociology, political sciences, geography): such dialogue is needed to gain updated insights of organised crime structures and activities.

Own elaboration. Given that the four types of OCGs are ideal–typical in nature, we accentuate and identify only the main ways through which each type shape space.

Finally, even this preliminary analysis suggests there is not any unique organisational model for organised crime groups (Catino 2019); it is therefore important to remember that as OCGs are presented as increasingly fluid and flexible networks, their relationship with space is not a given, and always needs to be taken into account when any research starts. Indeed, the territorial dimension, the space, and eventually the place, maintain their lasting relevance for comprehension of OCGs structures and activities even if one assumes an inclination towards the transnationality of organised crime activities.

## Special issue articles

In this article, we have shown the main processes through which OCGs can influence, create, transform spaces into places thus giving social meaning to territories. We have then highlighted how we can find patterns in the ways types of OCGs engage in specific processes of interacting with spaces. This theoretical framework is the starting point of this special issue on “Spaces of Organised Crime” and also the standard through which the papers have been elaborated and selected. We have followed a “maximisation of diversity” criterion: we have included papers dealing with different types of OCGs that are rooted in several areas of the planet. There are several *fils rouges* interconnecting the papers with one another, theoretically and critically. More importantly, they all contain robust empirical sections.

By matching the theoretical starting points to the empirical aspects, we notice the papers deal with the following themes, reflecting each the nexus OCGs and territories:

i. The interactions, the norms, the rituals, the behaviours of OCGs in physical spaces, ranging from ports, specific regions, urban contexts, local settings, border areas, or any other environment characterised by more or less stable boundaries.
ii. The manifestation of OCGs’ activities across a range of non-physical (virtual) spaces such as online forums, website on the deep/dark web or any “relational space” which is the result of network patterns.
iii. The interactions between OCGs and institutions such as the political and economic field or other complex more or less formalised fields characterised by bounded solidarity and cohesive trust rooted at the local level.

In all the papers, the space-shaping dimensions are present to different degrees, of course, depending on the OCGs’ structure and/or activity the paper is engaging with.

For example, *Marco Antonelli’s* paper, *“An exploration of organised crime in Italian ports from an institutional perspective. Presence and activities”,* engages with an extensive and diachronic perspective on the presence and activities of OCGs into Italian seaports in order to provide a systematic analytical map through the analysis of institutional law enforcement reports. In this paper, Antonelli looks at both mafia-type groups and criminal enterprises, different commodities and illicit activities, with a view to understand how the space of the port is exploited or is hindering these activities. According to the scheme we have proposed, we can postulate that Antonelli’s paper mainly look at how space in ports is diversified, represented, and partially controlled by OCGs. The institutional perspective of course formalises the space of the port and adds extra layers to the analysis.

Similar to Antonelli, *Robby Roks, Lieselot Bisschop*, and *Richard Staring* also look at the space of ports in their article *“Getting a foot in the door. Spaces of cocaine trafficking in the Port of Rotterdam”*. They focus on the port of Rotterdam and they base their article on a qualitative study, consisting of 73 interviews with public and private actors, an analysis of 10 criminal investigations, and field visits to public and private organisations in the port. They pay attention to those physical spaces in the port of Rotterdam that provide opportunities for cocaine trafficking and how criminal enterprises diversify activities in these spaces. Indeed, the current socio-spatial relations in the port of Rotterdam, the authors argue, also make the role of people on the inside increasingly indispensable, and this includes both public and private actors. OCGs, in the form of criminal enterprises, can not only diversity activities in the space of the port but also interconnect different actors and segments of their activities through the space as well. Diversifying and representing space within circumscribed areas are key analytical-issues in Roks, Bisschop and Staring’s work.

In *Vincenzo Scalia’s* article, *“The production of the Mafioso space. A spatial analysis of the sack of Palermo”,* the space analysed is the city of Palermo in Sicily, and the OCGs in question are mafia-type groups investing in construction and production of the urban space between the 1950s and the 1980s. What is called as ‘the Sack of Palermo’, the destruction of fertile land and the building of high-rise buildings, has been a vehicle for mafia-type groups in the city to both enrich themselves through contracts, but also to re-model the territory. Mafia-type OCGs in this article are actively controlling the space, they are allocating resources and shaping the activities in those places too. Their control over the urban space, the author argues, has been fundamental in the erosion of a collective sense of belonging in the city and made it to reinforce the domination of the mafia. By dealing with a mafia-type group, Scalia shows a poly-dimensional manipulation of space (see Table 1).

Also moving within an urban sphere and still remaining on mafia-type groups, is the article by *Martina Baradel* and *Jacopo Bortolussi*, *“Under a setting sun: the spatial displacement of the yakuza and their longing for visibility”*. In this article the authors reflect on how Japanese mafia-type OCGs, within the yakuza, use the spaces they occupy in Tokyo as part of their visibility tactic. They not only ‘control’ the space but also use it in its representing function. The space becomes place, for the yakuza, to establish power as well as to diversify their activities. Indeed, by using a case of a criminal group that strives for visibility, this article explores the relationship between OCGs, space and (in)visibility. Based on interviews and institutional documents, this article focuses on the wards of Kabukichō (Tokyo) and Nakasu (Fukuoka) – traditionally spaces of yakuza presence – examining how a politics of surveillance over urbanscapes and a spatial displacement of the yakuza induced a change in the yakuza’s relationship with their surroundings. This, the authors argue, has challenged the success of the yakuza in their criminal endeavours as well. Baradel and Bortolussi’s paper is, therefore, an original attempt to show representing space processes mixing exposure and concealment.

Remaining in the urban setting is the article by *Petr Kupka*, *Václav Walach* and *Alica Brendzová*, *“The poverty business: landlords, illicit practices and reproduction of disadvantaged neighbourhoods in Czechia”.* This article focuses on two cases of disadvantaged neighbourhoods: “the Hostel” in Brno and “the Neighbourhood” in Litvínov. The authors explore how regular neighbourhoods can decline as a result of illicit practices carried out by certain organised groups. The context of the paper is the so-called “poverty business”, which refers to the renting of overpriced, substandard housing, financed considerably using housing benefits and thus exploiting the housing need of vulnerable social groups, namely Roma. In this case, we have criminal enterprises at a local level that exploit the spaces, to control resources and shape them. In other terms, OCGs are mainly devoted here to diversify space and eventually to gain a window of opportunity to exert some forms of control.

The difference between the urban space and the rural space is at the core of the article by *Andy Clark*, *Alistair Fraser* and *Niall Hamilton-Smith*, *“Networked territorialism: the routes and roots of organised crime”*. The authors depart from an understanding of the network society, which fragments territorial space according to the logic of networked global capital. The authors elaborate the relationship between place, territory and criminal markets in Scotland. They compare an urban neighbourhood with a longstanding organised crime footprint and a rural community with a negligible organised crime footprint, where the drug economy is managed through a mobile criminal network based in England. In the rural space, criminal enterprises can play a role of interconnectivity based in low-risk, controllable territories with high-profit markets. In the urban space, the OCGs can diversify their activities because they have deep routes with the territory. Indeed, both communities demonstrated evidence of ‘networked territorialism’; the spaces do impact on the success and the management of criminal enterprises in those spaces. Through a comparative approach, Clark, Fraser and Hamilton-Smith’s paper shows distinctions between processes of diversifying and controlling space, on one side, and those of interconnecting space, on the other.

Moving towards a more nuanced idea of borders, the article by *Viviana García Pinzón* and *Jorge Mantilla*, “*Contested borders: organised crime, governance, and bordering practices in Colombia-Venezuela borderlands*”, looks at *borderland* as a spatial category in its own right. This, incidentally, also recalls the space of the ports in the previous papers, as both gates and doors to the sea. This paper looks at criminal enterprises in borderland, an institutionalised space characterised by practices and sovereignty between Colombia and Venezuela. The authors argue that criminal enterprises not only blur or erode the border but rather enforce it to their own benefit. Indeed, in this case we have apparently cross-border but de facto local criminal group in the borderland that control the space and use it to their advantage, they set norms to regulate socio-spatial practices, informal and illegal economies, and migration flows, creating overlapping social orders and, lastly, (re)shaping the borderland. García Pinzón and Mantilla’s paper shows a process of representing and controlling space, through which OCGs can also institutionalise themselves as key actors.

Last but not least, we have a paper by *Tim Hall, Ben Sanders, Mamadou Bah, Owen King* and *Edward Wigley*, *“Economic geographies of the illegal: the multiscalar production of cybercrime”*. Within economic geography, this paper understands the spatiality of illicit and illegal ‘industries’ as deriving from a different set of intellectual traditions for whom space is a less explicit, central concern. The authors explore the spatiality of one illegal industry, cybercrime, as a space for online criminal enterprises and fraud-related activities. It considers the spaces within which cybercrime is embedded, exploring it as the product of factors operating at multiple scales. The paper reflects on the potentials for the development of more spatially informed readings of cybercrime specifically, and illegal economic activities more generally. This paper speaks to the interactions, the norms, the rituals, the behaviours of OCGs in the online space as well as to the interactions between OCGs and institutions, specifically referring to the anti-cyber policing efforts and how they shape the online space. Again, we have a paper providing insights about the multidimensional link between OCGs and space, arising here in terms of soft/virtual processes of interconnection and control.

To conclude, we claim that the main aim of the special issue is to support and revamp investigations on the spatial aspects of organised crime phenomena. The relationship between OCGs and territories is bidirectional. On one side, OCGs shape space. On the other side, specific territorial areas with their (more or less) local resources, institutions, shared cultural orientations affect how OCGs operate. The papers selected for this special issue mainly deal with one of the directions of the relationship (OCGs- > Space). Still, they also suggest new venues for research on the other path (Space- > OCGs). Upon closer scrutiny, we recognise that this is a classic topic ever generating further research questions. In criminology, for instance, there is a long tradition of studies dealing with criminal phenomena unfolding in spatial niches. In sociology, the study of the spatialised forms of social dynamics dates back to Simmel's pioneering contributions. These historic literature streams are still counting for available analytical toolkits that we can apply alongside with innovative research techniques and concepts.

We find ourselves in a scenario characterised by conflicting tensions. Processes of territorial ‘atomisation’ (fragmentation) are growing, due to rising economic and social inequalities. At the same time, processes of territorial interconnections are increasing. The latter, however, can also suffer setbacks due to the emergence of shocking events, such as the Covid-19 pandemic. Against this background, there is no reason to doubt that the links between OCGs and territories will continue to pose new puzzles and questions for empirical research to explore.

## Footnotes

1 In this regard, the ideal types help overcoming two concerns that have monopolised – and in a certain way paralysed – the criminological debate on organised crime: ethnicity and the dichotomy ‘modernity-tradition’ (Sergi and Storti 2020). With respect to the ethnicity, it has long been conceived as a reified factor that could generate organised crime groups in terms of antagonist action (i.e. the alien conspiracy) or of in terms of mechanical adhesion to deviant norms (i.e. criminal subcultures). With respect to the dichotomy modernity-traditions, organised crime groups have been classified between old ones, which are involved in marginal business, and new ones, which are involved in dynamic economic sectors. Not recognising these assumptions, we have claimed that ethnicity can be conceived as an explicatory factor only if not depicted as independent variable: ethnic belonging is not a glue of communities. Rather, it is a label that organised crime groups can instrumentalise to rely upon. Furthermore, we have shown that any kind of organised crime groups is characterised by mixes between change and continuation of assets and activities.
